# Plasma microRNAs in human left ventricular reverse remodelling

**DOI:** 10.1515/med-2020-0179

**Published:** 2020-07-01

**Authors:** Rajan Rehan, Sanjay Patel

**Affiliations:** Royal Prince Alfred Hospital, Camperdown, New South Wales, Australia

**Keywords:** microRNA, heart failure, medical genetics, cardiac remodelling

Heart failure (HF) is a complex clinical syndrome in which the heart is unable to provide an adequate circulatory output to meet the physiological requirements of the body [1]. It can be further categorized according to left ventricular ejection fraction (LVEF), e.g. HF with reduced ejection fraction (LVEF < 40%) and HF with preserved ejection fraction (LVEF > 50%). The pathophysiological process is characterized by chronic neurohormonal activation that leads to cardiomyocyte dysfunction and fibrosis [2]. These changes can lead to maladaptive cardiac remodelling, a condition in which the molecular and cellular changes of the myocardium govern the macroscopic variation in structure and function. This self-perpetuating cycle leads to a further compromise in cardiac function and poor prognosis.

MicroRNAs (miRNAs) are a collection of 19–25 nucleotide endogenous, non-coding RNA molecules that act as post-transcriptional gene expression regulators and may potentially serve as novel diagnostic biomarkers or therapeutic targets [3,4]. Their functional role has been implicated within cardiovascular diseases including HF, arrhythmias and atherosclerosis. Previous studies have identified miRNA expression patterns in cardiac tissue and attempted to correlate individual miRNAs with specific disease processes. Nevertheless, there is limited evidence to support them as a biomarker for HF in clinical practice.

With a growing interest in this area, a recent study by Shah et al. (circulation) identifies a subset of miRNAs that predict left ventricular reverse remodelling (LVRR) in HF patients with reduced ejection fraction [5]. LVRR pertains to the regression of ventricular dilation and improvement in cardiac ejection fraction (defined as a 15% reduction in the end-systolic volume index in this study). This myocardial transformation is apparent in certain patients who respond to the neurohormonal blockade through the guideline-directed medical therapy. With emerging evidence demonstrating a correlation between LVRR and improved patient outcomes, there is now interest in identifying biomarkers to predict this phenomenon.

In this study, 64 participants from the PROTECT trial (Pro-BNP Outpatient Tailored Chronic HF Therapy: a prospective, randomized controlled trial of 151 patients with an LVEF < 40%) were identified and circulating miRNAs were prospectively measured from plasma samples. In total, 11 miRNAs were recognized to correlate with LVRR post-guideline-based medical therapy for approximately 10 months. Their target genes were subsequently identified using bioinformatics approaches. Furthermore, three of these miRNAs led to a significant downregulation of a set of targeted mRNAs, which were not previously determined in the pathogenesis of HF. The dysregulation of human miRNAs (miR-423, -212, -221 and -193b) alleviated the expression of targeted mRNAs via common pathways in both cellular and mouse models. The evaluation of these miRNA pathways showed an overlap of pathways consistent with myocardial hypertrophy and HF.

This study supports the evolving evidence that miRNA networks are crucial in the pathogenesis of HF. Moreover, it illustrates multiple plasma miRNAs associated with LVRR in a carefully phenotyped human cohort. The investigators utilized a multiplex circulating miRNA assay (FirePlex™) for quantification of miRNAs in the study. In general, multiplexing technologies such as the FirePlex™ system used by the investigators need to be validated on single-plex assays over another platform. The RNA isolation methodology should involve the use of synthetic spike-in miRNAs and the normalization/adjustment for any loss of sample during the processing workflow. Methodologies involving robotics and automation need to be introduced in the isolation of miRNAs or total RNA from biofluids and the coefficient of variation between users should be stated to assure the reproducibility of findings in subsequent studies.

As mentioned by the authors, study limitations include small sample size and inability to derive general conclusions given the absence of an external cohort. It also needs to be noted that most of the changes in levels of the miRNAs presented as relative to control are modest and that significant changes (>2-fold) in the levels of these miRNAs should be investigated using absolute quantitation approaches involving droplet digital PCR so as to understand the biological relevance of each miRNA. The FirePlex™ technology used by these investigators involves endpoint (rather than real-time) assessment of miRNA amplification. As indicated by the authors, a Pearson (rather than spearman) correlation of 0.74 was observed between the FirePlex™ and qPCR platform. Another limitation of the study, as discussed in the supplemental section of this study, is that all samples were analysed on a single assay plate. Future studies need to involve rigorous assessment of study samples to test batch variation, intra- as well as inter-user variation and provide more clarity in data presentation to facilitate reproduction of study findings by others in the field. It should be noted that technologies involving real-time PCR and droplet digital PCR should be used to assess transcripts that are of low abundance and yet relevant to vascular complications and disease.

Apart from these molecular/technological limitations, precision regarding the exact timing of echocardiographic follow-up could have potentially enhanced the association between miRNAs and LVRR. In addition, the animal investigation did not include genetic or pharmacological gain- and loss-of-function analysis of non-coding RNAs associated with LVRR. Theoretically, this may blur the exact role of these miRNAs in the pathogenesis of HF. CRISPR-based gene editing technologies could be used in future to assess the potential of these miRNAs as modulators/biomarkers of disease.

Moving forward, further clinical trials encompassing stringent endpoints and appropriate study designs are required to assess the potential utility of miRNAs in clinical practice.

**Figure 1:**
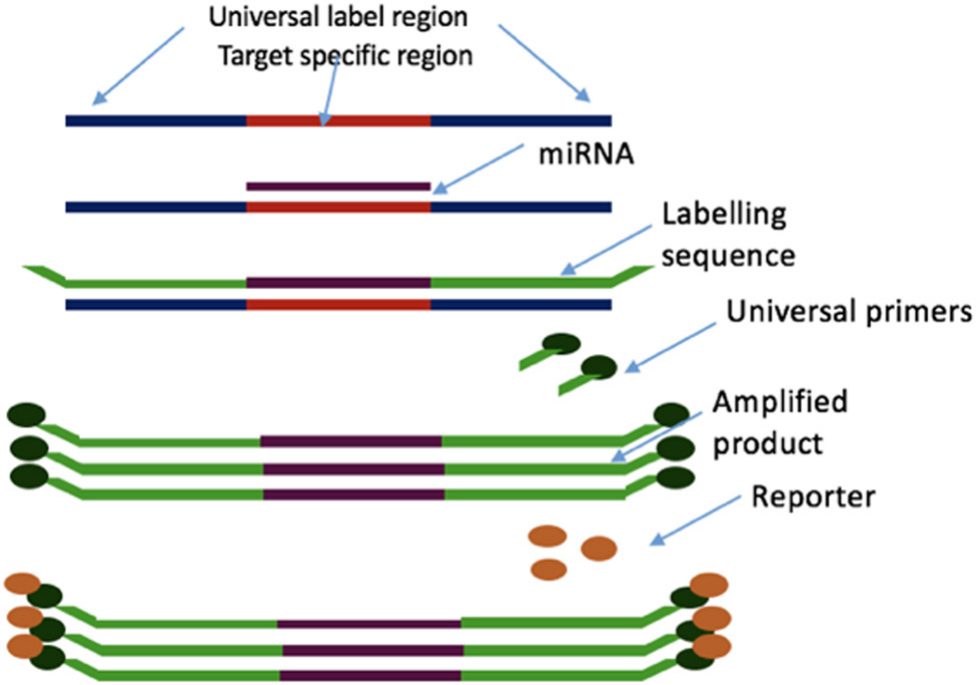
FirePlex™ microRNA assay. This allows the profiling of up to 65 target miRNAs of one’s choice simultaneously. It can be utilized to profile microRNAs directly from biofluids or purified RNA.

**Figure 2:**
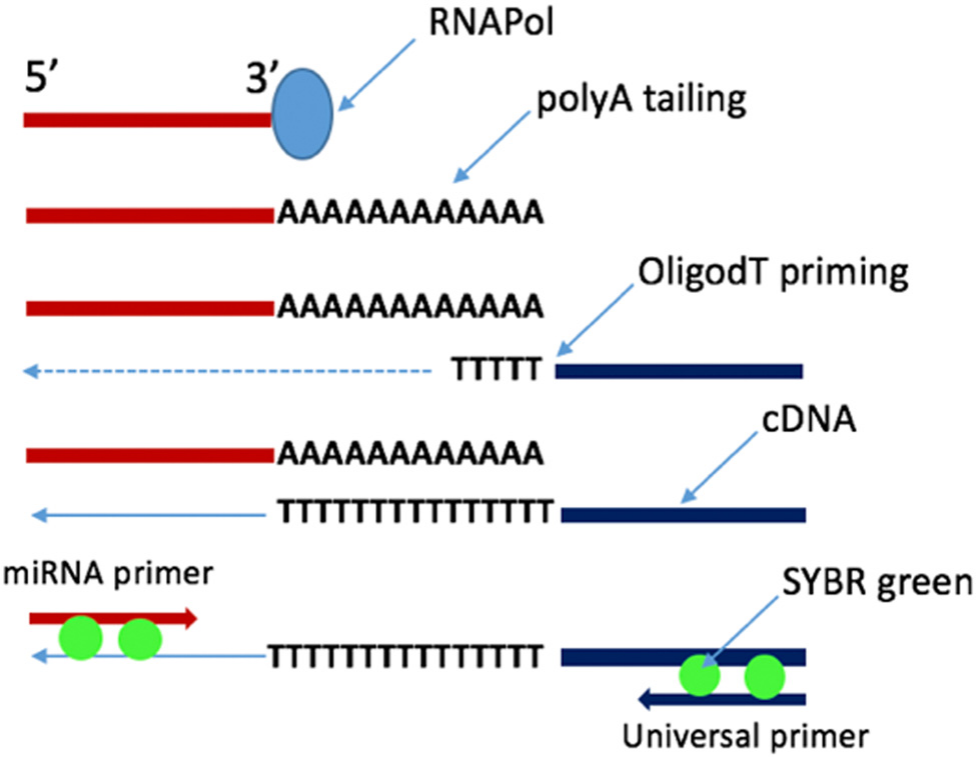
PCR-based miRNA quantification. This synthesizes a first stand cDNA from an RNA sample which is then amplified and quantified by qPCR using an miRNA-specific primer.

These should focus on a thorough recognition of corroborating changes in the myocardium and plasma of model systems with coherent results in humans. Precise identification of the cellular origin for these miRNAs will highlight the dominant tissues involved in the pathogenesis of HF. Overall, this study serves as a molecular discovery platform which enables further research into the extended role of miRNAs as a clinical biomarker, prognostic tool and potential therapeutic target (Figures 1 and 2).
